# A survey about laughter upon viewing functional seizures

**DOI:** 10.3389/fneur.2026.1725833

**Published:** 2026-02-03

**Authors:** Mohamad Z. Koubeissi, Nadim Jaafar, Christopher Saouda, Alexandra Eid, W. Curt LaFrance, Gülşen Öztosun, Hassna S. Aziz, Muhammad T. Khan, Faraaz A. Khan, Adam U. Syed, Adam Khalil, Candan Gürses, Tanvir U. Syed

**Affiliations:** 1 Department of Neurology, The George Washington University, Washington, DC, United States; 2 Departments of Neurology and Psychiatry, Comprehensive Epilepsy Program, Rhode Island Hospital, Brown Medical School, Providence, RI, United States; 3 Department of Psychiatry and Human Behavior, Rhode Island Hospital, Brown Medical School, Providence, RI, United States; 4 Neurological Institute, University Hospitals Cleveland Medical Center, Cleveland, OH, United States; 5 Charleston Area Medical Center, Charleston, WV, United States; 6 Ross University School of Medicine, Miramar, FL, United States; 7 University of Toledo Health Science Campus, Toledo, OH, United States; 8 Viterbi School of Engineering, University of Southern California, Los Angeles, CA, United States; 9 Koç University Hospital, Istanbul, Türkiye; 10 Access Telecare, Dallas, TX, United States

**Keywords:** behavior, clinical practice, functional seizures, laughter, nonepileptic seizures

## Abstract

**Introduction:**

Laughter among physicians when observing clinical manifestations of functional seizures (FS) or other functional disorders is frequently noted. This reflexive response can occur both in clinical practice and during video presentations at medical conferences. We examine the underlying factors contributing to physicians’ laughter in response to the diagnosis of FS.

**Methods:**

The research, spanning 5 years and diverse geographical locations, surveyed 221 participants, including physicians and non-physicians, to understand the reasons behind laughter during FS diagnoses.

**Results:**

A total of 221 respondents (estimated 20–25% of attendees) completed the survey, with 56% identifying as physicians and 44% as non-physicians. Observational data showed laughter responses to FS videos varied widely across settings: approximately 57% at U. S. medical grand rounds, compared to 5–17% at international conferences, and 0% among non-medical audiences. Survey analysis revealed 10 thematic categories for reasons behind laughter, with significant differences between physicians and non-physicians. Non-physicians more frequently cited defense mechanisms, negative opinions, and ignorance, whereas physicians more often attributed laughter to superiority, diagnostic skepticism, or perceived patient deception. U. S. physicians were significantly more likely than non-U. S. physicians to report discomfort, negative opinions, and ignorance. No significant differences were found between neurologists and internists.

**Significance:**

Laughter may serve multifaceted adaptive functions in response to the complexities of diagnosing and managing patients with FS. By highlighting misperceptions surrounding functional disorders, the study underscores the importance of fostering a deeper understanding among clinicians to ensure equitable care for patients experiencing FS.

## Highlights

- Many clinicians appear to perceive functional seizures (FS) as feigning symptoms.
- Laughter when viewing FS may be a defense mechanism and reflects ignorance about the seriousness of FS.
- Proper education of health care workers should help mitigate the laughter response to FS.

## 1 Introduction

Functional seizures (FS), previously known as psychogenic nonepileptic seizures as per International League Against Epilepsy (ILAE) definition, are complex neuropsychiatric disorders characterized by paroxysmal episodes that resemble epileptic seizures (ES) (1) and are associated with underlying psychological stressors and brain network connectivity abnormalities (2, 3). FS can be difficult to distinguish from ES without video-EEG monitoring (4), and patients experience prolonged durations of misdiagnosis and iatrogenic adverse effects (5, 6). While FS do not have electroencephalographic correlates (7, 8), emerging evidence suggests associated neurobiological mechanisms (9, 10).

Contrary to common misconceptions, FS represent a serious condition, and are the most common condition among all functional neurological disorders (FNDs). They are associated with substantial psychiatric comorbidities such as anxiety and depression, and increased risk of mortality (6, 11, 12, 31). The standardized mortality ratio for FS has been found to be 2–3 times greater than the general population, which is comparable to that of pharmacoresistant epilepsy (12). Some patients with FS present to hospitals with functional (nonepileptic) status, exposing them to invasive interventions, with associated risk of iatrogenic harm (13). Furthermore, undiagnosed FS and other FNDs are associated with significant financial burden to the individual and health care system (14). Conversely, their proper diagnosis and management have been shown to result in major reduction in the total seizure-related medical charges (15).

In the absence of focal neurological findings and a comprehensive neuropsychiatric approach, many neurologists and psychiatrists experience a disjunction in the approach to patients with FS (5, 16). The addition of the term “neurological” in DSM-5, which deemphasized psychological mechanisms in some interpretations, may have further influenced clinician attitudes and responses, including laughter, when encountering patients with FND. Physicians now often find themselves navigating a landscape characterized by ambiguities and constraints in their biomedical, reductionistic communication to patients and other specialties (17), fostering a somewhat cynical attitude toward their patients with psychological issues. Conversely, patients grapple with the burdensome implications of the skepticism of the psychopathological processes associated with the diagnosis, or the absence thereof, leading to ongoing empiric treatment with unhelpful antiseizure medications for presumed epilepsy, which can prove to be an overwhelming experience (7, 18, 19).

In educational symposia on FS, including ones at the annual meetings of the American Epilepsy Society and the American Academy of Neurology, seizure monitoring unit videos of seizures in patients with FS have often been observed as followed by laughter among some audience members. This raised the question: “Why do clinicians laugh when viewing FS?.” In this study, we aim to examine whether physicians and non-physicians laugh at the display of FS and their perceived reason behind laughing, assessing potential implications of this behavior on patients and public opinion. While Tolchin et al. (20) characterized joking about FS as a maladaptive defense mechanism among clinicians—suggesting that such humor may alleviate discomfort while reinforcing stigmatizing perceptions—empirical research on laughter as a clinician response to patient symptoms remains limited.

## 2 Methods

In an effort to assess real-time, anonymous surveillance of reactions to seizure videos in educational settings, over a period of 5 years, two epileptologist authors (MZK and TUS) presented lectures on the etiology, diagnosis, and treatment of FS at several medical and non-medical meetings and gatherings in the US and abroad. In all lectures, we included videos showing functional convulsions, drop attacks, speech arrest, and unresponsiveness.

### 2.1 Samples

The medical audience consisted of attendees of Epilepsy and Neurology Grand Rounds (USA), International Epilepsy Symposium (Marburg, Germany), Epilepsy Course (São Paulo, Brazil), Turkish Epilepsy Congress (Bodrum, Turkey), and Internal Medicine Grand Rounds at Case Western Reserve University in Cleveland, OH. The non-medical audience consisted of candidates for master’s degree in Ethics at Case Western Reserve University in Cleveland, OH, USA and Freshman Undergraduate Sociology classes at Edinboro University, PA, USA.

### 2.2 Measures

With, by design, the audience being unaware of being observed, the speaker and an unannounced member in the audience visually counted the audience that laughed while watching the FS videos. The same videos were shown to all audiences in this study. The audience was not observed closely for laughter while watching epileptic seizures, when shown in presentations. A written question was administered after the talk to all audience members (including those who laughed) to be answered in free text. The question was: “*Why do you or others laugh, giggle, or smile when observing FS or psychogenic non-epileptic seizures?*.” Participants were not asked for any personal information other than their specialty or field of study. We did not inquire about the years of experience in the specialty or prior exposure to/or knowledge of FS. Two investigators, a physician and a non-physician, conducted a Thematic Analysis (TA) of the free text responses, generating a thematic list of the subject-reported reasons for laughter. This analysis followed the widely accepted six-phase framework proposed by Braun and Clarke (21). For each reason, the inter-rater reliability was calculated. Discrepancies in classification, if any, were to be resolved by a third investigator, a physician (Table 1).

**Table 1:** Rigorous methodological steps supporting the validity of thematic analysis.

Phase of thematic analysis	Description of implementation
Familiarization	Both investigators (physician and non-physician) independently read and re-read all free-text responses.
Generating initial codes	Each investigator independently highlighted and labeled segments of text with codes related to the explicit and implicit reasons for laughter.
Searching for themes	The investigators met to compare their initial codes, grouping similar codes together to form preliminary themes (potential reasons for laughter).
Reviewing themes	The investigators reviewed the themes against the entire dataset to ensure they accurately reflected the meanings in the data (internal homogeneity and external heterogeneity).
Defining and naming themes	Consensus was reached on the final set of themes, and clear, descriptive names were assigned.
Producing the report	The themes were used to structure the findings, with supporting quotes (exemplars) provided to illustrate each one.

### 2.3 Statistical analysis

We used descriptive statistics to summarize participants’ characteristics and answers (the main outcome measures). Categorical variables are presented as percentages, and Chi-square tests were used to calculate *p*-values.

## 3 Results

**Figure 1:**
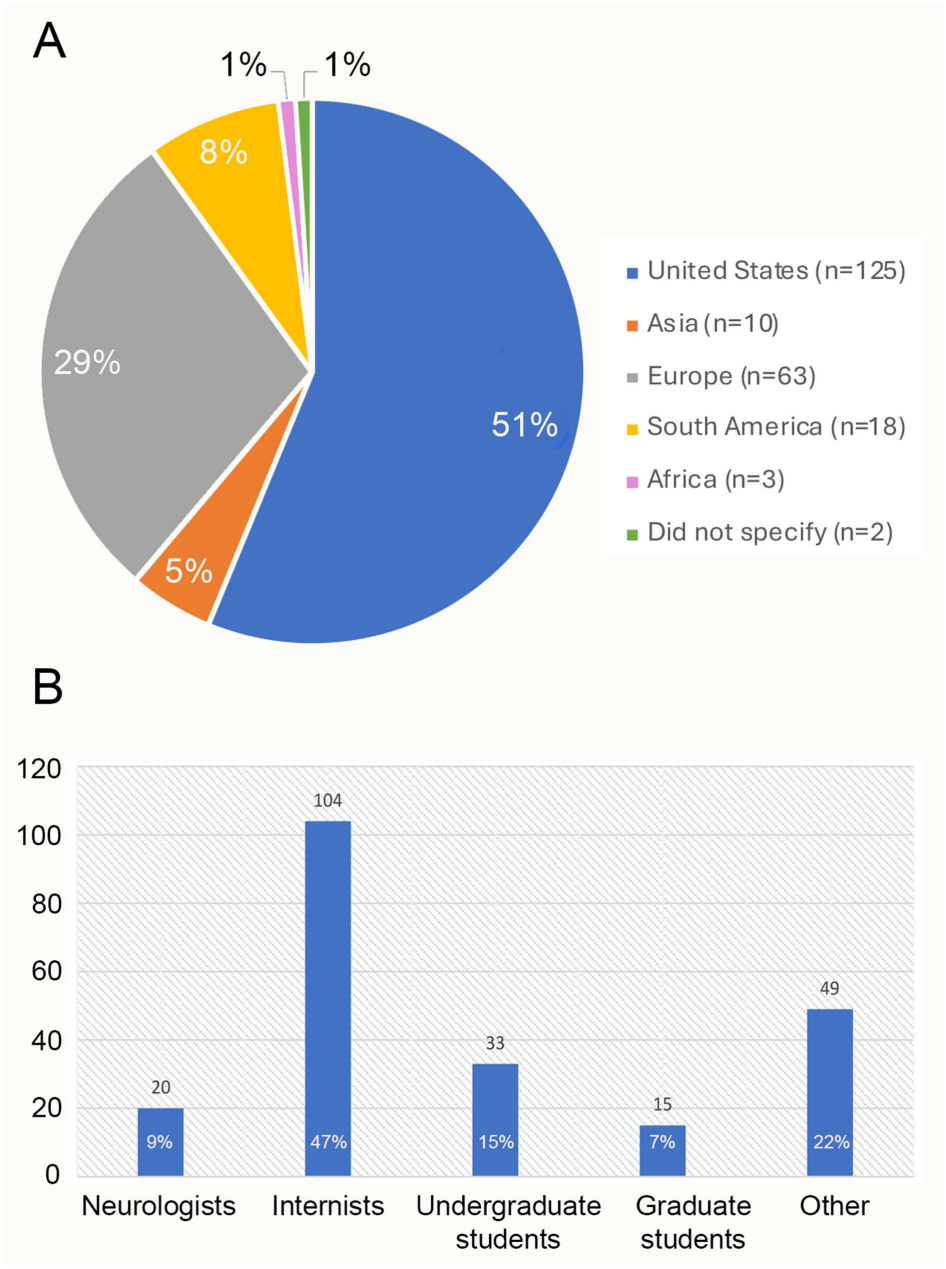
The geographical locations of the respondents **(A)** and the distribution of their specialties **(B)**.

Of all approached subjects, 221 filled out responses to the question. The total number of attendees at these conferences was not documented, but an approximately estimated 20–25% of attendees responded. It was not possible to keep track of what particular categorical subsets of these viewers laughed in response to FS videos. The locations of the respondents are summarized in Figure 1A. Among these subjects, 124 responders (56%) were physicians and 97 (44%) were non-physicians. Of note, while the medical conferences would have included medical students, all responders identified as physicians without specifying if they were attending physicians or trainees. The specialties of the respondents are shown in Figure 1B, where the category “Other” includes students of animation, anthropology, bioethics, journalism, business marketing, ceramics major, communication and media studies, computer studies, criminal justice, graphic design, health and physical education, history, and human services (Figures 1A,B).

As previously discussed, the first part of the study involved observing the attendees at different medical lectures to determine the percentage of each audience that responded to the FS videos with laughter. This number was found to be approximately 57% at epilepsy grand rounds, general neurology grand rounds, and internal medicine grand rounds in the US, ~5% at an epilepsy symposium in Marburg, Germany, ~8% at an epilepsy course in Sao Paolo in Brazil, ~13% at the Turkish Epilepsy Congress in Bodrum, Turkey, and ~17% in the remaining countries. None (0%) of the non-medical audiences in either the ethics or sociology classes were observed to respond with laughter.

Each respondent was allowed to include more than one reason for laughter in the surveys, though most respondents only listed one reason. Their responses were divided into 10 categories, designated as: *Category 1*, “Defense mechanism/discomfort,” as to denote that the subject explained the laughter as a defense mechanism due to a discomfort (“nervous laughter”); according to this reason, laughter would basically be a displacement behavior triggered as an automatic behavioral reaction to reduce an unhandleable discomfort. Laughter would be an emotional coping response. *Category 2*, “Superiority/Arrogance/Pride/Cannot fool me,” denotes that the laughter observed in this context arises from a sense of superiority on the part of the observer, who believes that the patient cannot deceive or mislead them. *Category 3*, “Negative Opinion (immature, unprofessional, cruel, sadistic),” implies that the subject’s laughter falls into this category due to behaviors characterized by immaturity, unprofessionalism, cruelty, or sadistic tendencies. *Category 4*, “Not organic,” encompasses situations where the clinician either does not express concern about the diagnosis or does not initially consider the presence of a disease. *Category 5*, “Faking,” denotes instances where the surveyed individuals believe that either the patient is intentionally pretending or that the physician has mistakenly interpreted the FS as an act of deception on the patient’s part. *Category 6*, “Ignorance,” represents situations where the response is rooted in a lack of knowledge or understanding.

*Category 7*, designated as “Relief/Event is benign,” signifies that the laughter stems from the relieving perception that the situation or event is harmless or non-threatening. According to this reason, laughter reflects a relief that is mediated by cognitive appraisal that the event is benign.

*Category 8*, “Semiology (funny, bizarre, theatrical),” encompasses scenarios where the patient’s behavior is seen as eccentric or dramatic, leading to laughter. *Category 9*, “Other,” indicates that the respondent had a different, unspecified reason for their reaction (e.g., “the clinicians who laugh themselves have a neurological disorder,” or they “do not know how to react”). Finally, *Category 10*, “No answer,” denotes instances where there was no response or explanation provided. The inter-rater reliability was near-perfect for generating this thematic list of the subject-reported reasons for laughter.

A comparative analysis of selections between physicians and non-physicians across several categories revealed statistically significant differences (Tables 2, 3). Categories 1 (Defense mechanism/discomfort), 3 (Negative opinion – immature, unprofessional, cruel, sadistic), and 6 (Ignorance) were significantly more common among non-physicians. Conversely, categories 2 (Superiority/Arrogance/Pride/Cannot fool me), 4 (Not organic), and 5 (Faking) were significantly more common among physicians. There were no statistically significant differences in categories 7, 8, 9, and 10. Categories and examples are provided in Tables 2, 3.

**Table 2:** Category of responses to the question “Why do you or others laugh, giggle, or smile when observing FS or psychogenic non-epileptic seizures?” and examples of responses.

Survey categories	Category name	Examples
Category 1	Defense mechanism/discomfort	“Uncomfortable reaction.” “May be because people sometimes laugh when they are uncomfortable.” “Nervous laughter - I did not know how to respond appropriately.”
Category 2	Superiority/Arrogance/Pride/Cannot fool me	“It is unbelievable that the patient believes that they can trick the doctor, esp. the epileptologist.” “Because now I can figure out what is the nature of the events that have fooled the family and other caregivers. Somehow like a police catching the neck of a thief.” “The sophistication of a psychogenic seizure is correlated with the patient’s IQ. The more obvious seizure comes from patients with low intelligence. This person is like a child lying, thinks she can fool a brilliant adult like me.”
Category 3	Negative opinion (immature, unprofessional, cruel, sadistic)	“Unprofessional, lacks interest in helping the patient.” “Ignorance or it reminds the [laughing viewers] of something they have seen before. This is not professional.” “The person may be a young or immature individual who does not think the seizures are real or who just finds them funny.”
Category 4	Not “organic”	“Maybe because we can figure out that it is not a real seizure.” “Most people see it as a psychological thing, not a medical problem.” “I never really thought about this, but maybe it is because we think that the situation and episode is not real organic disease.”
Category 5	Faking	“It looks fake.” “It is obviously a nonepileptic event and visualizing the event and thinking about the great effort being made to fake an event makes you giggle.” “Because it appears foolish for someone to “try to fool” others.”
Category 6	Ignorance	“I do not laugh. I think people laugh because they cannot understand that psychogenic events are also a result of brain processing.” “They are most likely laughing because of the lack of knowledge about what is happening.” “They do not understand the concepts of what is behind a person having such a seizure and they think it’s funny.”
Category 7	Relief/Event is benign	“When I identify them as psychological events, not medical, so they do not warrant ER.” “Because of the knowledge of no seizure activity and that it is not a potential life-threatening event.” “Everyone feels that outcome is going to be good.” “Because it seems that you finally realize it is not an epileptic seizure, your feel relief and you see it with different eye.”
Category 8	Semiology (funny, bizarre, theatrical)	“Because it is a little bit funny to see how patient make things to simulate an event that does not exist.” “It’s funny, and sometimes I think that person is copying someone’s behavior (not conscious) but is funny.” “Videos of PNES often appear bizarre and strange.”
Category 9	Other	“We giggle but we should not.” “Because they feel uncomfortable since they or someone they know has that same problem.” “I do not know why it happens.”
Category 10	No answer	

**Table 3:** Category of response among physicians and non-physicians.

Survey categories	Category name	Physician (*n* = 124)	Non-physician (*n* = 97)	*p*-value
Category 1	Defense mechanism/discomfort	4%	23%	<0.001
Category 2	Superiority/Arrogance/Pride/Cannot fool me	14%	3%	<0.001
Category 3	Negative opinion (immature, unprofessional, cruel, sadistic)	1%	21%	<0.001
Category 4	Not “organic”	13%	1%	<0.001
Category 5	Faking	29%	14%	<0.01
Category 6	Ignorance	5%	14%	<0.01
Category 7	Relief/Event is benign	9%	4%	0.037
Category 8	Semiology (funny, bizarre, theatrical)	19%	13%	0.118
Category 9	Other	5%	7%	0.411
Category 10	No answer	1%	0%	0.472

Comparing the responses of physicians based on country of origin, USA physicians were more likely to express category 1 (Defense mechanism/discomfort, 87%), category 3 (Negative opinion, 93%), and category 6 (Ignorance, 82%), than non-USA physicians (13, 7, 8%, with *p*-values of <0.001, <0.001, and <0.01, respectively). Among the non-medical audiences, there were no clinically significant differences between the answers of undergraduate and graduate students. Interestingly, none of the non-medical audiences expressed the category “Not organic.” Table 4 shows the differences in the responses of neurologists and internists, where no statistically significant difference was found between the raters in any category.

**Table 4:** Numbers and percentages of occurrences for each identified category of the reasons for laughter among neurologists and internists.

Categories	Category 1	Category 2	Category 3	Category 4	Category 5	Category 6	Category 7	Category 8	Category 9	Category 10
Neurologists, *N* (%)	1 (5)	5 (25)	0 (0)	3 (15)	9 (45)	1 (5)	3 (15)	2 (10)	2 (10)	4 (20)
Internal Medicine, *N* (%)	7 (7)	18 (18)	2 (2)	17 (17)	36 (35)	6 (6)	13 (13)	28 (27)	7 (7)	17 (17)

N, number of respondents.

## 4 Discussion

This survey is the first to explore clinicians’ perspectives on laughter when viewing seizure videos from patients with functional seizures (FS). Respondents, including both clinicians and non-clinicians, reported on their own emotional and cognitive reactions after laughing at the videos or, if they did not laugh themselves, provided insights into why they believed others did. The sample was diverse and cross-cultural, comprising over 100 physicians from four continents (North America, South America, Asia, and Europe) and nearly 100 non-clinicians.

One of the most commonly perceived reason for laughter among clinicians was the misconception that patients were faking their symptoms. This misconception likely arises from the fact that FS lacks electroencephalographic signatures and the clinicians being unaware of associated neurobiological correlates in FS (22–24). This misconception may also be influenced by stigma, which is prevalent in functional seizures and is often reinforced by indirect forms of clinician bias or dismissive attitudes (25).

Moreover, laugher is a known defense mechanism stemming from the discomfort or embarrassment of dealing with FS per clinician respondents (20). Another prominent reason of laughter when dealing with patients with FS is the relief due to the perceived ease of treatment when compared to the alternative, ES. Others laughed when not taking FS or functional disorders seriously. It is unclear why a higher percentage of participants appeared to laugh in the US than in other countries. One could posit that there are cultural differences across societies in their respective countries. According to Ramachandran (26), the laughter that is brought by relief is in a sense, nature’s way of telling us that everything is alright, and this is just a false alarm. When laughter and “eye rolling” are displayed on rounds, it may be a coping manifestation, as a reflection of the powerlessness we experience when we, as physicians, who have treatment algorithms for status epilepticus and code stroke, are rendered impotent with a patient whose shaking we cannot make stop. In the pedagogical realm, these behaviors are strong potential influencers of our colleagues, teams, and students, shaping a dismissive attitude toward a suffering patient population. When patients experience dismissive attitudes for their diagnosis, patients may move away from their condition in search of more diagnostic testing, which can lead to more expense, iatrogenesis, confusion and distrust of clinicians.

Laughter and stress are often viewed as opposite poles in affect regulation, with the laughter-associated positive affect being stress-buffering (27). With the *relief theory* of laughter; however, one could argue that there are more similarities with stress than meets the eye. Laughter, stress, anxiety, and most emotions could both be thought of as manifestations of underlying nervous energy. To that end, laughter could be motivated by multiple factors. For example, our study shows that laughter upon viewing of FS could originate from a superior point of view for some clinicians, putting patients under scrutiny for faking their symptoms. In addition, the “unusual” symptoms that are incongruent with classical ES findings and the relief of the diagnosis being “easy” to treat could all be present at the same time.

When we assessed the responses of the non-physicians, who constituted 44% of respondents (graduate and undergraduate non-medical students), they also had their stance on the issue. They reported similar reasons to why providers laugh at FS diagnosis, however, they added that FS semiology may be perceived funny by clinicians or that clinicians cannot be fooled by what is “not real.” This idea harkens back to the former pejorative term, “pseudoseizures,” which is not used any more, as *pseudo* implies false or fake seizures. As explained to patients, FS are real seizures, even if they are not caused by epileptiform activity (4). Most non-physicians expressed disapproval of the physician’s unjustified laughter. The lack of laughter among non-physicians may be attributed to the alarming seizure presentation, as well as the perceived emotional stress and physical trauma in patients with FS. This is obviously in addition to the fact that they lack knowledge about what differentiates epilepsy from FND. On the other hand, laughter may be a coded way of physicians demonstrating to their peers that have the knowledge base to suspect this is not epilepsy. The perceived reasons of laughter point toward a lack of clinicians’ awareness of the implications of FS on the patient’s safety and life. By definition, FND occur unconsciously, and patients are not motivated by conscious gains. This debunks the misconception of patients faking their symptoms or trying to seek attention by them. Furthermore, papers by Nightscales et al. (28) and Reuber et al. (13), mentioned earlier, emphasize the morbidity of FS in terms of physical, iatrogenic, and psychological risks, as well as mortality. The misconception that functional somatic symptoms are innocuous stems from a longstanding disregard for the significance of comprehensive mental health education and a tendency to underestimate the gravity of disorders with psychological underpinnings and comorbidity. Ultimately, these misperceptions can influence clinicians’ attitudes toward their patients, potentially impeding the delivery of equitable care, regardless of the clinicians’ personal beliefs and perspectives.

In the pursuit of upholding the Hippocratic Oath and safeguarding patients’ well-being, we, along with others (29), contend that medical training should prioritize a deeper focus on FNDs. In all videos presented, there was no gelastic semiology before, during, or after the FS events; therefore, infectious laughter originating from the patients themselves could not account account for the observed audience responses. Although identical videos were shown to all viewers, we did not formally quantify laughter frequency in relation to specific FS semiologic features or perceived “bizarreness.” Correlations between laughter and particular semiologic characteristics therefore cannot be reliably assessed, and this adds onto the list of limitations of the study. On the other hand, while groupthink may contribute to the higher laughter rates observed in U. S. medical settings, the lower rates internationally and absence among non-medical audiences suggest a substantial role for cultural and professional norms.

The response of clinicians toward the diagnosis of FS has many implications on patients and the general population. Laughing at patients’ symptoms may reflect stigma to the patient and their family members. Not acknowledging the diagnosis as serious often leads to tension and a strained doctor-patient relationship, conceivably exacerbating the severity of the condition and potentially distancing patients from clinicians and the health care that could be provided. In addition to knowledge gaps, the nature of this type of stigmatization in patients with FS and related functional neurological disorders frequently lead to patients being dismissed or delegitimized by healthcare professionals, which is known to be associated with poorer quality of life and systemic negative attitudes in clinical settings; acknowledging that such stigma can shape clinician responses underscores the need for improved education and empathy in clinical practice (30).

This study has a number of limitations. First, the responders were not necessarily the attendees that reacted with a laughter to the viewing of FS. Accordingly, the responses may not reflect the opinion of attendees who respond to FS with a laughter. This reflects a problem in the survey question itself, for laughing subjects are likely to provide a more “justified” explanation of their laughter. Additionally, although the concordance between observers in estimating laughter responses was consistently near-perfect, these counts were not formally documented, preventing calculation of inter-rater reliability metrics such as the kappa statistic, and the reported percentages are reported as, and should be considered, approximations. These limitations may introduce minor uncertainty in the reported observational data and should be interpreted accordingly. Other limitations include overrepresentation of USA, inability to fully differentiate participants by years in practice and type of practice, the lack of standardization for “percentage of room laughter.” Another limitation is that we did not inquire how much training / knowledge the responders had on the topic, which may impact the interpretation of understanding of FS.

In addition to the above, phrasing the question as “Why do you or others laugh, giggle, or smile when observing FS?,” by including “others,” leads to the possibility that participants may be making false assumptions as to why others laugh. As such, the true reasons for why laughter occurred may be underrepresented and presumed reasons for laughter may have been overrepresented. Participants may try to make themselves look better, and others less so. Another limitation is asking respondents why a spontaneous behavior occurs, as it could elicit an artificial construct of explaining emotions.

## 5 Future recommendations

To build on this first of its kind study, future studies could investigate reasons for laughter when viewing FS, and could include level of training and understanding of FS to examine impact of education. Moreover, they could use questionnaires that include questions on the emotional response to viewing a FS of the clinician with several choices to select from (e.g., semiology is funny; feeling impotent at the lack of understanding of these seizures, etc.). As in the present study the videos were presented by speakers from a stage to a group of persons, there is the possibility that guessing the right diagnosis may have been perceived as a collective game, and that laughter stemmed from the self-perceived success in identifying the correct clinical condition. To better investigate this aspect, a future study could present the videos to individual subjects and to groups, and evaluate if the percentages of laughter differ between these two conditions. We also did not systematically quantify laughter in relation to specific FS semiologies or perceived unusualness, which may limit our ability to assess whether particular clinical features preferentially elicited laughter and highlights an area for future investigation. Based on direct observation and post-viewing interviews, however, we observed that laughter typically emerged once viewers recognized the functional nature of the event. Systematic analysis of semiology-specific viewer responses would be valuable to study further. Additionally, future studies could directly test expectation bias by presenting identical seizure videos with different diagnostic labels (epileptic vs. functional) to determine whether laughter is driven by preconceived notions of FS rather than by seizure semiology itself.

In conclusion, healthcare clinician education and increased research, clinical interest, and funding in functional disorders, such as FS, motor FND, and the like, may improve the patient-physician interaction, and this familiarity could potentially reduce morbidity, mortality, and financial burden of patients with FS and other FND. To better understand clinician behaviors attitudes, controlled mixed-methods studies may provide further insights into “why doctors laugh” when viewing FS videos.

## Data Availability

The original contributions presented in the study are included in the article/supplementary material, further inquiries can be directed to the corresponding author.
